# Menstrual hygiene management knowledge, practice and associated factors Among School Girls, Northeast Ethiopia

**DOI:** 10.1371/journal.pone.0271275

**Published:** 2022-07-19

**Authors:** Zeru Shikur Shumie, Zinie Abita Mengie

**Affiliations:** 1 Department of Obstetrics and Gynecology, Saint Peter Specialized Hospital, Addis Ababa, Ethiopia; 2 Department of Reproductive Health, College of Medicine and Health Science, Mizan-Tepi University, Mizan Aman, Ethiopia; IIPS: International Institute for Population Sciences, INDIA

## Abstract

**Background:**

Women in rural settings particularly in schools suffer more from stigma and lack of services and facilities during menstruation. However, the issue has not received proper attention from school water sanitation and hygiene programs. And this study was aimed to identify knowledge and practice of menstrual hygiene, and associated factors.

**Method:**

Institution based cross-sectional study was employed among 441 school girls in Mekidela city. EpiData Version 4.6 and the Statistical Package for the Social Sciences Version 25.0 were used for data entry and analysis, respectively. Bivariable and multivariable logistic regression was employed to identify factors associated with the outcome variables. Variables with a p-value<0.25 in the bivariable analysis were a candidate for multivariable logistic regression and p-value<0.05 in the multivariable analysis was used to declare significant association.

**Results:**

Of the respondents, 64.9% reported good knowledge of menstrual hygiene management. In multivariable analysis; grade level (grade 11 & 12) [adjusted odds ratio (AOR) = 2.23, 95% C.I (1.19–4.16)], age ≥ 19 years [AOR = 3.45, 95% C.I (1.78–6.69)], residence [AOR = 1.90, 95% C.I: (1.12–3.23)], know sanitary pads [AOR = 2.67, 95% CI: (1.19–6.00)] and learned about menstrual hygiene [AOR = 1.71, 95% CI: (1.02–2.87)] found to be associated with knowledge. About 62.4% have good menstrual hygiene practices. Knowledge on menstrual hygiene [AOR = 1.73, 95% C.I (1.07–2.80)], know about reproductive tract/sexually transmitted infections [AOR = 2.46, 95% CI: (1.37–4.43)], having private shower [AOR = 2.04, 95% C.I 2.04(1.24–3.37)] and residence [AOR = 3.76, 95% C.I:(2.18–6.51)] were factors associated with practice.

**Conclusion:**

Overall nearly two-third of respondents had good knowledge and good practice of menstrual hygiene management. Factors significantly associated with their knowledge included age, grade level, residence, learning on menstrual hygiene, and knowledge of sanitary pads. Residence, knowledge on menstrual hygiene, know about sexually transmitted infections and having a private shower were found to be associated with practice of menstrual hygiene management. In general, our finding indicates that menstrual hygiene was unsatisfactory among adolescent school girls and more should be done on the factors identified.

## Introduction

Menstruation is the periodic losing of the internal lining of the uterus via the vagina alongside blood beneath Neath the manage of the hormones of the hypothalamus-pituitary-ovarian axis [1]. Menarche, or the onset of menstruation, is a landmark function of girl puberty and alerts reproductive maturity. Women start menstruating at the average age of 13 and preserve menstruating till the common age of 51. The menstrual cycle is commonly round 28 days however can range from 21 and 35 days. The bleeding lasts between two to seven days with some days lighter and some heavier bleeding. The cycle is commonly abnormal for the first two years after its onset [2–4].

Menstrual hygiene management refers to the use of clean materials by girls and women to retain menstrual blood, which can be changed stealthily, safely, hygienically, and as often as necessary during menstruation. Managing menstruation is dealing with menstrual blood flow as well as continuing regular activities like going to school, playing and dancing, doing routine activities, etc. [5].

Generally, women have their strategies to cope with menstruation with great variation from country to country, and within countries, depending on an individual’s personal preferences, available resources, economic status, local traditions, and cultural beliefs and knowledge or education [6]. However due to different cultural and social misconception in association with menstruation, most adolescents girls do not have sufficient information on how to maintain their menstrual hygiene [7].

For girls to have healthy, productive, and dignified lives, they must be able to manage menstrual bleeding effectively. To achieve this, it is essential for girls to have access of appropriate WASH facilities, private room to change their sanitary material, waste disposal site for the used one and access to information about menstrual cycle and how to manage it hygienically [8].

The unhygienic exercise of menstruation can raise the incidence of reproductive tract infection (RTI). Thus, the consequences of RTIs are severe and may result in a significant negative impact on a woman’s health including pelvic inflammatory disease, dysmenorrhea (painful periods), and in severe cases infertility. Approximately every year 10% of women worldwide are exposed to genital infections including bacterial vaginosis, urinary tract infections and 75% of women have a history of a genital infection [9].

Although poor knowledge and unsafe menstrual hygiene practices have significant clinical implications for girls and their future offspring, adolescent girls’ knowledge of menstruation is poor and their practice is unhygienic, in particular in developing countries [10]. Evidences are showing that between 40% and 45% of school-age adolescent girls have poor knowledge and unsafe hygiene practices of their menstrual bleeding [11]. One study in Ethiopia found that 68.3% of adolescents have poor knowledge on menstrual hygiene management. Not having learned about menstruation at school, rural residency and lower grade level were some of the factors associated with poor knowledge on MHM [12]. This could have clinical implications for integrating the promotion of menstrual hygiene practice into the health care system and global efforts are needed including policies to improve girls’ knowledge and safe hygiene practices towards menstruation.

Globally, according to World Health Organization (WHO) report, 2.3 billion girls and women didn’t manage their menstruation safely due to lack of facilities for MHM, high cost and ignorance. As a result their lonely option is to use old clothes, or other unhygienic materials as menstrual absorbents, which may impose them to infections and other related health consequences [13].

The evidence from United Nations International Children’s Emergency Fund (UNICEF) revealed that 10% of school-age African girls miss school during their monthly bleeding [14]. Unavailability of WASH facilities at school is the primary reason that influence the attendance of girls in school and as most of girls are unable to afford sanitary pads and they use insanitary rag. And they, frustrate, become anxious and miss class due fear of development of bad odor [15].

In our country Ethiopia, around 17% of girls miss school during their menstruation with a roughly equal proportion among urban and rural girl. The repeatedly listed reasons for the absenteeism are discomfort, fear of having an accident at school, embarrassment, and lack of sanitary material to manage their period on site. The likelihood of absenteeism is also seemed to vary based on the girls’ usual ways of MHM [16].

In Ethiopia, one study revealed that 25% of girls do not use any sanitary material to manage their menstruation and prefer to isolate themselves during menstruation. The evidence also shows that only 25% of girls have informed about MHM at school [17].

The menstrual hygiene management Policy and Implementation Guideline of Ethiopia call for engagement of stakeholders at different level of government structures from federal to kebele level. With this continuum of actors, the guidelines propose a multi-sectoral approach that includes health, water and irrigation, education, women and children’s affairs, labor and social affairs, civil society organizations. The guideline focuses on four major components including awareness creation, availing WASH facilities, Supply and provision of sanitary pads and Management and disposal of used sanitary materials. However, awareness and hygienic practice among adolescent girls is still a problem.

Despite of the crucial role of safe menstrual hygiene practices in helping millions of women with related complications and complex problems, it is still a missed opportunity to address understanding and hygienic practice of menstruation among pre-adolescent girls in many developing countries, including Ethiopia [18,19]. And it is an unrecognized and unaddressed public health problem in WASH research in developing countries such as Ethiopia. Few studies available in Ethiopia have not particularly focused on the knowledge or practices of menstrual hygiene separately and associated factors addressed so far were limited to sociodemographic one. Here in our study we have included school related factors which could have a potential influence on menstrual hygiene. Therefore, this study was intended to assess the level of menstrual hygiene knowledge, practices, and associated factors among secondary school girls in Mekdela town. The outcome of this study could be of critical clinical importance and could help plan, prepare policies and develop appropriate intervention mechanisms.

## Methods and material

### Study design and setting

An institution-based cross-sectional study was employed in Mekdela district which is one of the districts in the South Wollo zone, and its capital city is Masha located 553 km away from Addis Ababa, 635 km from Bahirdar, and 152 km from Dessie. In Ethiopia currently there is a three-year project on MHM by WASH Flagship that sponsored by MADEBLUE Company of Netherland. The project primarily aimed to contribute to enhanced health and well-being of girls and women by promoting gender equality, rights, and reducing barriers to girl’s education. Mekdela secondary school is the only secondary and preparatory school in the Mekdela district. currently, the school has a total of 1707 female students from grade 9^th^ to grade 12^th^. The study was conducted from March 05 to March 15, 2020.

### Population

All school girls who have reached menarche and were attending their education in Mekidela secondary schools were the source population. And the study population was secondary school girls who met the eligibility criteria. All girls from grades 9th to 12th who were attending their education on the regular program were included in the study.

### Sample size determination

The sample size was calculated using single population proportion formula with the following assumptions:—A 95% confidence interval, 5% margin of error, and 51.36% female adolescent students had good knowledge about menstruation and its management from the previous study [17], considering 10% non-response rate the calculated sample size is 422. Whereas, 52.5% of students had good menstrual hygiene practice among adolescent school girls in the previous study [20], considering a 10% non-response rate the calculated sample size is 441. Therefore, the final the largest sample size(441 was taken) for the representativeness issue.

### Sampling procedures

A stratified sampling technique was used to select participants from all grades. The first sample was proportionally allocated to each grade level by the number of female students. Then finally a simple random technique was used to recruit the desired samples by using the student list as a sampling frame.

### Study variables

The outcome variables were knowledge and practice of menstrual hygiene. The independent variables were socio-demographic and economic factors; knowledge, and source of related information; sanitary pad-related factors; and WASH-related factors.

### Operational definition

#### Good knowledge of MHM

Knowledge about MHM was measured by 8 items. Each correct answer earned 1 point and each incorrect response was scored as 0. The mean score of menstrual knowledge was computed and used as a cutoff point to classify [21]. Those who scored greater than or equal to the mean score value were considered as having good knowledge [22].

#### Poor knowledge of MHM

Those who scored less than the mean score value were considered to have poor knowledge [22].

#### Good practice of MHM

Menstrual hygiene practice was measured by 11 items. Each correct response earned 1 point and each incorrect response was scored as 0. Accordingly, the mean score was used to decide the cutoffs of the rank [21]. Those who scored greater than or equal to the mean score value were considered as having good practice [22].

#### Poor practice of MHM

Those who scored less than the mean value was considered to have poor knowledge [22].

### Data collection tool and procedure

Data were collected by using pretested structured self-administered Amharic version questionnaires adapted from a review of related literature and prepared as recommended by UNICEF for assessing MHPs [20–22]. It consists of four sections (socio-demographic information/ economic/ family-related, knowledge about menstrual hygiene management related, menstrual hygiene practice-related, and school or environmental related questions). Self-administered questionnaires were distributed to the students by four trained female midwives with experience in data collection, and the data was collected in classrooms and two female teachers were facilitating the orientation and dissemination of the questionnaire. Finally, the filled questionnaires were checked for completeness and consistency of the data by the data collector’s supervisors and principal investigators.

### Data quality management

The quality of data was assured at the maximum attainable level by using a structured questionnaire adapted after reviewing related literatures and following the necessary procedures to get the intended results. To ensure the quality of data, the pre-test was done on schoolgirls in Addis Ababa Entoto Amba secondary school by taking 5% of the total sample size. Data collectors and supervisors have orientation one day before data collection on the objectives of the study and data collection procedure by the principal investigator. The questionnaire was checked for completeness and correctness daily by immediate supervisors.

### Data analysis

The data was cleaned, coded, and entered into Epi-data version 4.6 software, and then exported to Statistical package for social sciences (SPSS) version 25 for statistical analysis. Descriptive analysis was used to describe the major characteristics of the respondents. Bivariable logistic regression was used to identify the association between the independent and the dependent variables. Those variables with a p-value < 0.25 in the bivariable analysis were a candidate for multivariable logistic regression, and then those variables with a p-value<0.05 in the multivariable analysis was declared as having statistically significant association with menstrual hygiene management practice. Multicollinearity was checked by using VIF and not detected (VIF<10). Hosmer and Lemeshow goodness of fit test was used to assess the model fitness (χ^2 =^ 7.4866, df = 8, p-value = 0.485)

### Ethical approval and consent to participate

Ethical clearance was obtained from the Institutional Review Board of Addis Ababa University College of Health sciences. Written requests to conduct the study were made to the Mekdela district education office and the permission to conduct the study was granted by Mekdela secondary and preparatory school. School directors and directresses were briefed on the objectives of the study and permission to conduct the study was obtained from participating schools. Informed consent was obtained from each study participant and assent from parents/guardians was taken for those with age <18 years.

## Results

### Socio-demographic characteristics of the study population

A total of 441 female secondary school girls were participated in this study, with a response rate of 100%. Most of the participants,176 (n = 324,73.5%) were from grades nine and ten, and the majority of them,225 (51%) came from the urban area. The mean age of the study participants was 17.72 with SD ± 1.487 years, while more than three forth of them were in the age group from 15–18 years. Majority of adolescent girls, 310(72.1%) experience their first menstruation in the age range of 12–14 years.

More than half of the respondents 243 (55.1%) were Muslim followers followed by 186 (42.2%) and 12 (2.7%) orthodox and protestant followers respectively. Regarding education status, 178 (40.4%) of the respondents’ mothers had no formal education and 145(39.2%) of their fathers had no formal education. One hundred ninety-four (44.0%) respondents’ mothers were housewives and 202 (45.8%) of their fathers were farmers. Two hundred eighty-two (63.9%) respondents’ parents have a private shower. Three hundred twenty-three (73.2%) respondents get permanent pocket money from their families. Majority (92.3%) of study participants are single in marital status and more than half (56.7%) of respondents are living with their both parents (Table 1).

**Table 1:** Participants Socio-demographic, family-related characteristics in Mekdela secondary school girls, Amhara Regional State, Northeast Ethiopia, 2020 (n = 441).

Variable	Response category	N	%
Age of respondents	15–18	331	75.1%
	>/ = 19	110	24.9%
Menarcheal age	12–14	318	72.1%
	>/ = 15	123	27.9%
Grade	9–10	324	73.5%
	11–12	117	26.5%
Residence	Urban	225	51.0%
	Rural	216	49.0%
Religion	Orthodox	186	42.2%
	Muslim	243	55.1%
	Protestant	12	2.7%
Marital status	Single	407	92.3%
	Married	34	7.7%
With whom do you live	Both parents	250	56.7%
	Mother only	55	12.5%
	Relatives	35	7.9%
	Father only	38	8.6%
	Other (specify)	63	14.3%
Educational status of the father	No formal education	145	32.9%
	Primary	116	26.3%
	Secondary	86	19.5%
	College and above	94	21.3%
Educational status of a mother	No formal education	178	40.4%
	Primary	152	34.5%
	Secondary	75	17.0%
	College and above	36	8.2%
Occupation of father	Farmer	202	45.8%
	Merchant	88	20.0%
	government employee	81	18.4%
	private organization employee	16	3.6%
	daily laborer	35	7.9%
	Driver	19	4.3%
Occupation of mother	Housewives	194	44.0%
	Farmer	92	20.9%
	Merchant	81	18.4%
	Private organization employee	13	2.9%
	Government employee	33	7.5%
	Daily laborer	28	6.3%
Parents provide money regularly	Yes	323	73.2%
	No	118	26.8%
Parents have a private shower	Yes	282	63.9%
	No	159	36.1%

### Knowledge about menstrual hygiene management

Only 3 participants scored 0 out of eight items. Two hundred eighty-six (64.9%) of the participants had good knowledge about MHM. Three hundred forty-three (77.8%) of girls perceive that menstruation was a physiological process, and 47 (10.7%) of the girls believed that it was a curse from God. Nearly half of the respondents (n = 210, 47.6%) knew that the cause of menstruation is hormonal, and more than half, 300 (68.0%) of the respondents knew that uterus is the origin of the menstrual blood. Three hundred eight (69.8%) responded that the normal menstrual bleeding duration is 2 to 7 days, and 168 (38.1%) of girls believed the normal duration of the menstrual cycle is 20–35 days. The majority of the respondents 399 (90.5%) knew that the availability of sanitary pads in the market and 324 (73.5%) knew about reproductive tract infections or sexually transmitted infections. Only 201 (45.6%) of the respondents have discussed menstrual issues with their parents and friends. More than half of the respondents, 306 (69.4%) got information about menstruation before attaining menarche (Table 2).

**Table 2:** Respondents Overall Knowledge about menstrual hygiene management in Mekdela secondary school, Amhara regional state, Northeast Ethiopia, 2020 (n = 441).

Variable	Response category	N	%
Menstruation is	Is a physiological process	343	77.8%
	Is a pathological process	19	4.3%
	I curse from god	47	10.7%
	I don’t know	32	7.3%
Cause of menstruation	Hormone	210	47.6%
	Is caused by sin	43	9.8%
	It is a curse of God	84	19.0%
	Is caused by a disease	9	2.0%
	I don’t know	95	21.5%
Source of menstrual bleeding	Uterus	300	68.0%
	Vagina	79	17.9%
	Abdomen	42	9.5%
	I don’t know	20	4.5%
Normal menstrual bleeding duration	< 2 days	55	12.5%
	2–7 days	308	69.8%
	>7 days	45	10.2%
	I don’t know	33	7.5%
Normal duration of the menstrual cycle	< 20 days	93	21.1%
	20–35 days	168	38.1%
	>35days	101	22.9%
	I don’t know	79	17.9%
Menstruation is a lifelong process	Yes	151	34.2%
	No	290	65.8%
Menstrual blood is unhygienic	Yes	358	81.2%
	No	83	18.8%
Foul-smelling during menstruation	Yes	374	84.8%
	No	67	15.2%
Heard about menstruation before menarche	Yes	306	69.4%
	No	135	30.6%
Do you know about RTIs/ STIs	Yes	324	73.5%
	No	117	26.5%
Do you know sanitary pads in the market	Yes	399	90.5%
	No	42	9.5%
Discuss menstrual issues with your parents, friends?	Yes	201	45.6%
	No	240	54.4%
Level of knowledge about MHM	Good knowledge	286	64.9%
	Poor knowledge	155	35.1%

### Factors Affecting knowledge of menstrual hygiene management

In the bivariable analysis, some of the socio-demographic and other menstrual-related characteristics of the respondent show association with the outcome variable-knowledge about menstrual hygiene management.

Under multivariable analysis respondents’ age, grade, area of their residence, know sanitary pad in the market and learn about MHM in school showed significant association with knowledge about MHM. Students whose age (≥ 19 years) were 3.45 times more likely to have good knowledge on menstrual hygiene management than their counterparts who were 15–18 age groups [AOR = 3.45, 95% C.I:(1.78–6.69)]. On the other hand, students of grade eleven and twelve were 2.23 times more likely to have good knowledge of menstruation and its hygienic management than those [AOR = 2.23, 95% C.I: (1.19–4.16)] of grade nine and ten. In addition to this, the odds of good knowledge on MHM were 1.90 times higher among girls who came from urban areas than those who reside in rural areas [AOR = 1.90, 95% C.I: (1.12–3.23)]. Girls who know sanitary pads in the market were 2.67 times more likely to have good knowledge about menstruation and its hygienic management than their counterparts who don’t know sanitary pads in the market [AOR = 2.67, 95% CI: (1.19–6.00)] (Table 3). Girls who have learned about MHM in the school were 1.71 times more likely to have good knowledge about menstruation and its hygienic management than those who have not learned.

**Table 3:** Factors independently associated with knowledge on menstrual hygiene management of Mekdela secondary school girl students, Amhara region, Northeast Ethiopia 2020.

Variable	Response category	Knowledge		COR (95% CI)	P.val	AOR (95%CI)	P.val
		Poor (N, %)	Good (N, %)				
**Age**	15–18	138(41.7%)	193(58.3%)	1		1	
	>/ = 19	17(15.5%	93(84.5%)	3.91(2.23–6.86)	0.001	3.45(1.78–6.69) *	0.001
**Grade**	9 & 10	136(42.0%)	188(58.0%)	1		1	
	11 & 12	19(16.2%)	98(83.8%)	3.73(2.18–6.39)	0.001	2.23(1.19–4.16) *	0.012
**Residence**	Urban	59(26.2%)	166(73.8%)	2.25(1.51–3.34)	0.001	1.90(1.12–3.23) *	0.018
	rural	96(44.4%)	120(55.6%)	1		1	
**Discuss about menstruation with their parents, friends**	yes	52(25.9%)	149(74.1%)	2.15(1.44–3.23)	0.001	1.39(0.81–2.41)	0.24
	No	103(42.9%)	137(57.1%)	1		1	
**Know sanitary pad in the market**	yes	125(31.3%)	274(68.7%)	5.48(2.72–11.06)	0.001	2.67(1.19–6.00) *	0.017
	No	30(71.4%)	12(28.6%)	1		1	
**Learned about MHM in the school**	yes	82(28.4%)	207(71.6%)	2.33(1.55–3.51)	0.001	1.71(1.02–2.87) *	0.041
	No	73(48.0%)	79(52.0%)	1		1	
**Parents have a private shower**	Yes	80(28.4%)	202(71.6%)	2.25(1.50–3.38)	0.001	1.41(0.85–2.35)	0.188
	No	75(47.2%)	84(52.8%)	1		1	

NB: Significant at *P-value<0.05.

### Practice towards menstrual hygiene management

Most of the participants (n = 275, 62.4%) of the respondents had good menstrual hygiene practice. From four hundred forty-one study participants, 241(54.6%) use commercially made disposable sanitary pads though 86(19.5%), 85(19.3%) use reusable sanitary pads, and disposable pieces of rags respectively. Fifty-eight of the girls (13.2%) changed used pad only once per day whereas 19(4.3%) changed three times and above per day. 171(38.8%) wash their genitalia area with only water. On the other hand, 138(31.3%) of the respondents dispose of their used menstrual pads in the bin. The majority of the respondents, 261(59.2%) do not take a bath at all during the menstrual episode. The majority, 331(75.1) of respondents wash their reusable sanitary pad with soap and water (Table 4) Nearly half of the respondents 200(45.6%) didn’t use commercial made sanitary pads, the reason was high cost 103(23.4), followed by lack of knowledge 44(10%), unavailability 37(8.4), shyness 16(3.6) respectively.

**Table 4:** Respondents Overall practice on menstrual hygiene management in Mekdela secondary school, Amhara regional state, Northeast Ethiopia, 2020 (n = 441).

Variable	Response category	N	%
**Do you use sanitary material**	yes	441	100.0%
**what sanitary material use**	Disposable sanitary pads	241	54.6%
	Disposable piece of rags	85	19.3%
	Reusable sanitary pads	86	19.5%
	Paper/toilet paper	0	0.0%
	Underwear	29	6.6%
**reason for not using a disposable sanitary pad**	Lack of knowledge	44	10.0%
	High cost	103	23.4%
	Unavailability	37	8.4%
	Shyness	16	3.6%
**wash your genitalia during menstruation**	yes	308	69.8%
	no	133	30.2%
**what medium do you use**	Only Water	171	38.8%
	Soap and water	138	31.3%
**how often wash your genitalia per day**	once	38	8.6%
	twice	168	38.1%
	thrice	67	15.2%
	>/ = four times	36	8.2%
**Do you take bath during menstruation**	yes	180	40.8%
	no	261	59.2%
**how often take bath during menstruation per day**	< = Two times in a day	109	24.7%
	> Two times in a day	72	16.3%
**Do you change your sanitary material**	yes	308	69.8%
	no	133	30.2%
**How often change absorbent material per day**	once	58	13.2%
	twice	148	33.6%
	thrice	83	18.8%
	more than three times	19	4.3%
**How do you dispose of menstrual materials**	Open field	0	0.0%
	latrine	303	68.7%
	Put in the bin	138	31.3%
**Where store your new or reusable absorbent**	Drawers	17	3.9%
	Dress cabinet	105	23.8%
	Bathrooms	76	17.2%
	Store with routine cloth	239	54.2%
	I don’t store it	4	0.9%
**What materials are used for Washing reusable cloth**	With soap and water	331	75.1%
	With water only	110	24.9%
**where keep your reusable pads after washing for drying**	In the shade outside	33	7.5%
	In the shade inside	129	29.3%
	In the sunlight inside	55	12.5%
	In the sunlight outside	52	11.8%
	hidden under other clothes	143	32.4%
	hidden elsewhere	29	6.6%
**Level of Practice about MHM**	Good practice	275	62.4%
	Poor practice	166	37.6%

### Factors Affecting menstrual hygiene practice

In the bivariable analysis, some of the socio-demographic and other menstrual-related characteristics of the respondents were significantly associated with the outcome variable-practice of menstrual hygiene management.

Table 5 below showed that, the association between participants’ overall characteristics and menstrual hygienic practice. In the multivariable analysis, respondents’ area of their residence, knowledge about MHM, parents having a private shower, know about RTIs / STIs show statistically significant association with student’s menstrual hygiene practice at 95% CI and 0.05 level of significance.

**Table 5:** Factors independently associated with menstrual hygiene practice of Mekdela secondary school girl students, Amhara region, Northeast Ethiopia 2020.

Variable	Response category	Practice		COR (95% CI)	P.val	AOR (95%CI)	P.val
		Poor (N, %)	Good (N, %)				
**Residence**	urban	38(16.9%)	187(83.1%)	7.16(4.60–11.14)	0.001	3.76(2.18–6.51) *	0.001
	rural	128(59.3%)	88(40.7%)	1		1	
**Knowledge about MHM**	Good	84(29.4%)	202(70.6%)	2.70(1.80–4.05)	0.001	1.73(1.07–2.80) *	0.025
	Poor	82(52.9%)	73(47.1%)	1		1	1
**Educational status of a mother**	No formal education	90(50.6%)	88(49.4%)	0.12(0.04–0.36)	0.001	0.79(0.24–2.64)	0.706
	Primary	59(38.8%)	93(61.2%)	0.20(0.07–0.59)	0.003	0.84(0.26–2.72)	0.764
	Secondary	13(17.3%)	62(82.7%)	0.60(0.18–1.98)	0.398	1.02(0.30–3.54)	0.974
	College and above	4(11.1%)	32(88.9%)	1		1	
**Parents having a private shower**	Yes	69(24.5%)	213(75.5%)	4.83(3.18–7.34)	0.001	2.04(1.24–3.37) *	0.005
	No	97(61.0%)	62(39.0%)	1		1	
**Heard about menstruation before menarche**	Yes	92(30.0%)	214(70.0%)	2.82(1.86–4.28)	0.001	1.03(0.55–1.93)	0.933
	No	74(54.8%)	61(45.2%)	1		1	
**Know about RTIs/ STIs**	Yes	91(28.1%)	233(71.9%)	4.57(2.91–7.16)	0.001	2.46(1.37–4.43) *	0.003
	No	75(64.1%)	42(35.9%)	1		1	
**Know sanitary pads in the market**	Yes	136(34.1%)	263(65.9%)	4.84(2.40–9.74)	0.001	1.70(0.73–3.94)	0.216
	No	30(71.4%)	12(28.6%)	1		1	
**Discuss menstrual issue with their parents and friends**	Yes	41(20.4%)	160(79.6%)	4.24(2.77–6.50)	0.001	1.24(0.69–2.20)	0.476
	No	125(52.1%)	115(47.9%)	1		1	

NB: Significant at *P-value<0.05.

Students who had good knowledge about menstruation and its hygienic management were 1.73 times more likely to practice good menstrual hygiene than their counterparts [AOR = 1.73, 95% CI:(1.07–2.80)]. On the other hand, students living in urban areas were 3.76 times more likely to practice good menstrual hygiene than those who live in rural [AOR = 3.76, 95% C.I:(2.18–6.51)]. Girls who know about RTIs / STIs were 2.46 times more likely to practice good menstrual hygiene than those who don’t know about RTIs /STIs [AOR = 2.46, 95% CI: (1.37–4.43)]. In addition to this, students with parents having private showers were 2.04 times more likely to have good menstrual hygiene practice than those whose parents have not private shower [AOR = 2.04, 95% C.I: (1.24–3.37)] (Table 5).

## Discussion

This school-based study was aimed to examine adolescent girls’ knowledge and practice on menstrual hygiene management and have tried to identify sociodemographic, information related, and WASH factors associated with it.

Our study finding indicates that 64.9% of school girls have good knowledge managing menstrual hygiene. The finding is in line with another study conducted in Ethiopia [21]. And most adolescent girls (69.4%) had been informed about menstruation before menarche mainly from their mothers (36.3%) followed by teachers (26.3%). This result is similar to the finding conducted in Boset district, East Shoa, Oromia region, Ethiopia where 72.8% of girls had been informed about menstruation before its onset and their mother (36.5%) and teachers (32.4%) were their primary source of information [23].

Our study also found that 62.4% of girls have a hygienic practice in managing their menstruation. This prevalence is lower compared to finding from a previous similar study conducted in Ethiopia [3]. The possible reason could be study participants from a previous study were more likely to have access to facilities needed to manage menstruation and, their teachers being their main source of information may increase their chance of getting appropriate and adequate information on menstruation. Here the implication is that providing adequate information on menstrual hygiene at school can help adolescent girls to practice safe menstrual hygiene management.

Around 55% of girls have used sanitary pads available on the market. It is lower compared to the finding from a previous study in Ethiopia [21]. The variation might be due to socioeconomic differences between them. And 33.6% of the girls changed pads two times per day during their menstruation. The finding is in line with the study done at Mehalmeda (34.8%) [3].

Regarding factors associated with girls’ knowledge of MHM, five factors have been identified from our multivariable analysis. Being an urban resident is found to be significantly associated with having good knowledge about menstrual hygiene management. This is consistent with other studies conducted in the Amhara region and Bose district, Oromia region in Ethiopia [23,24].The possible reason could be those who live in urban are more likely to be exposed to information disseminated through media about menstruation and menstrual hygiene. It is indicative that awareness creation programs and interventions MHM didn’t address rural residents as they do for urban one.

The study also found that students who attended grades eleven and twelve were 2.23 times more likely to have good knowledge of menstruation and menstrual hygiene. This is consistent with the finding from Boset district, Oromia region [23]. This might be because girls learn more about menstruation and its hygienic management as their level of education increases. Here the implication is our finding is emphasizing existing shreds of evidence and it is crucial to include MHM as a school health program.

The age of girls was the other factor associated with their knowledge of menstrual hygiene. Those aged ≥19 are more likely to have good knowledge than the youngers. This finding is in agreement with the study conducted in India that reported that elder adolescents have statistically better knowledge about menstruation as compared to their peers in the early adolescent group. The possible explanation for such association might be elders experience menstruation more and are likely to seek information about it without being shy from different sources [25]. The finding is indicating that early adolescents are not getting adequate information on menstruation and its hygienic management.

Having learned about menstruation at school was also found to be associated with good knowledge of menstrual hygiene management. This is consistent with the study conducted in Adama town Ethiopia [26]. This might be due to that they have understood the physiologic nature of menstruation and have a clue on how to manage it. The main point here is that information from formal or informal sources is very critical for girls to have an understanding of such biological phenomenon in their life cycle.

Knowledge of sanitary pads available on market was the other factor that show association with menstrual knowledge. Those who know sanitary pads are 2.67 times more likely to have good knowledge to manage menstruation. The possible reason could be they may have the same source of information simultaneously for both sanitary pads and about MHM. Here the finding is implying that factories and sellers should promote on awareness and how to manage menstruation at same time they do for their products.

Our study also found four factors that have an association with MHM practice. Among them, Knowledge about MHM is one predictor of the practice of MHM. this is supported by findings from other studies [21,26]. This could be because if they know what to do, it will be easier for them to practice correctly. It implies that policies and programs should focus first on improving adolescent girls’ awareness of menstrual hygiene.

Having a private shower (access to WASH facilities) is the other factor associated with good practice of girls’ menstrual hygiene management. The finding is consistent with other studies conducted in Amhara region which found that gilrs who have access to WASH facilities are more likely to practice good menstrual hygiene than those who didn’t have [3,21]. The possible reason could be water supply is an important input when thinking about hygiene and sanitation issues. The implication from the finding is that policies and programs in the area of WASH should be comprehensive and multipurpose.

The practice of menstrual hygiene management has also a significant association with the area of residence. It is consistent with findings from other studies [21,27–29]. This might be due to the fact that girls who live in urban areas are at a higher chance of getting information on menstrual hygiene management, and accessing sanitary pads available on market. The finding is confirmatory that girls in the rural areas need special support in managing menstruation considering sociocultural barriers impeding them to do that.

Knowing about STI /RTIs is the other factor found to be associated with menstrual hygiene practice. This might be due to the interrelatedness of both issues that interventions targeting one may at the same time advocate on the other. It is implying that programs on STI prevention should address all the possible sources of infection with substantial focus as well on the menstrual hygiene management process.

Overall this study identified the level of knowledge and practice in managing menstruation which is almost in agreement with previous local and global findings. And the factors associated with the outcome variables were consistent with other findings indicating appropriate interventions has not been implemented.

### Limitation of the study

Due to the cross-sectional nature of the study, there was difficult to establish a causal relationship between the dependent and the associated factors. Further longitudinal studies are encouraged to address these gaps. Due to the sensitivity of issue, there will be higher tendency of social desirability bias. To compensate it we have tried to minimize by using female data collectors and female teachers to facilitate the data collection process. There was also a recall bias to get the exact age of menarche. To minimize it we used to link their first menstruation with the grade level they were attending by that time.

## Conclusion

Overall the study found that nearly two-thirds of respondents have good knowledge and practice of menstrual hygiene management. And factors associated with knowledge about MHM were the age of participants, grade level of respondents, learning about menstrual hygiene in the school, area of their residence, and know sanitary pads in the market. Regarding the practice of menstrual hygiene, knowledge about MHM, knowledge about RTIs/ STIs, the residence of the respondents, parents having a private shower (access to WASH facilities) were the factors associated with menstrual hygiene practice.

## Supporting information

S1 Appendix Amharic (local) version of the questionair. A survey on Menstrual Hygiene management knowledge, practice and associated factors Among School Girls, Northeast Ethiopia.
(DOCX)

S2 Appendix English version of the questionair. A survey on Menstrual Hygiene management knowledge, practice and associated factors Among School Girls, Northeast Ethiopia.
(DOCX)

## Abbreviations

AOR: Adjusted Odds Ratios
COR: Crude Odds Ratio
IRB: Institutional Review Board
CSA: Central Statistical Agency of Ethiopia
MHM: Menstrual Hygiene Management
NGO: Non-Governmental Organization
PI: Principal Investigators
RTIs: Reproductive Tract Infections
SNV: Sanitary napkin vendor
SPSS: Statistical package for social sciences
SRS: Simple random sampling
UNICEF: United Nations International Children’s Emergency Fund
WASH: Water, Sanitation and Hygiene
WHO: World Health Organization
