# Supplementary material for: Menstrual hygiene management knowledge, practice and associated factors Among School Girls, Northeast Ethiopia
Source: PLoS One. 2022 Jul 19;17(7):e0271275. doi: 10.1371/journal.pone.0271275 (PMC9295965; doi:10.1371/journal.pone.0271275)
Supplement: S1 Appendix — A survey on Menstrual Hygiene management knowledge, practice and associated factors Among School Girls, Northeast Ethiopia. (DOCX) [file pone.0271275.s001.docx]

Annex VIII የአማርኛ ቃለመጠይቅ

| **ክፍል አንድ፤ የተጠያቂዉ አጠቃላይ የማህበራዊ፣ ኢኮኖሚያዊና ቤተሰባዊ መረጃን በተመለከተ** | | | | | |
| --- | --- | --- | --- | --- | --- |
| **ተ.ቁ** | | **ጥያቄ** | | **መልስ** | **ዝለል** |
| 101 | | አሁን እድሜዎ ስንት ነው? | | እኔ---------አመቴ ነው |  |
| 102 | | የትምህርት ደርጃ | | 1.=9ኛ 2. =10ኛ  3.=11ኛ 4. =12ኛ |  |
| 103 | | የመጀመሪያ የወር አበባ ሲያዩ ዕድሜዎ ስንተ ነበር? | | እኔ ------ ዓመት ነበርኩ |  |
| 104 | | መኖሪያ ቦታ | | 1. ከተማ  2. ገጠር |  |
| 105 | | ሃይማኖት | | 1. ኦርቶዶክስ  2. ሙስሊም  3. ፕሮቴስታንት  4. ካቶሊክ  5. ሌላ (ይግለጹ) ----- |  |
| 106 | | የጋብቻ ሁኔታ | | 1 ያላገባች  2 የፈታች  3 ባል የሞተባት  4 ያገባች  5 የማይተገበር |  |
| 107 | | የአኗኗር ሁኔታ | | 1.ከሁለቱም ወላጆች  2. ከእናቴ ብቻ  3. ከዘመዶች  4. ከአባቴ ብቻ  5. ሌላ ካለ ይግለጹ) --- |  |
| 108 | | የእናት የትምህርት ደረጃ | | 1. መደበኛ ትምህርት ያልተማረች  2. የመጀመሪያ ደረጃ  3. ሁለተኛ ደረጃ  4. ኮሌጅ እና በላይ |  |
| 109 | | የአባት የትምህርት ደረጃ | | 1. መደበኛ ትምህርት ያልተማረ  2. የመጀመሪያ ደረጃ  3. ሁለተኛ ደረጃ  4. ኮሌጅ እና በላይ |  |
| 110 | | የእናት የሥራ ሁኔታ | | 1. የቤት እመቤት  2. ተማሪ  3. ነጋዴ  4. የግል ድርጅት ሠራተኛ  5. የመንግሥት ሠራተኛ  6. የቀን ሰራተኛ  7. ሌላ (ይግለጹ) ------ |  |
| 111 | | የአባት የሥራ ሁኔታ | | 1. ገበሬ  2. ነጋዴ  3. የመንግሥት ሠራተኛ  4. የግል ድርጅት ሠራተኛ  5. የቀን ሰራተኛ  6. ሹፌር  7. ሌላ (ይግለጹ) --- |  |
| 112 | | ወላጅዎ የኪስ ገንዘብ በቋሚነት ይሰጥዎታል? | | 1. አዎ  2. የለም | ለ ጥያቄ ቁጥር 112 መልስዎ የለም ከሆነ ወደ 114 ይሂዱ |
| 113 | | ለጥያቄ ቁጥር 112 መልስዎ "አዎ" ከሆነ የወር ገቢዎ ስንት ነው? | | 1. </= 200  2. 200-400  3. >/= 400 |  |
| 114 | | ወላጅዎ ቤት ውስጥ የግል ገላ መታጠቢያ አለ? | | 1. አዎ  2. የለም |  |
| **ክፍል 2 - ስለወር አበባ ንጽህና አጠባበቅ እውቀት መለኪያ ጋር የተያያዙ ጥያቄዎች** | | | | | |
| 201 | | \| የወር አበባ ምንድነዉ? \| \| --- \| | | 1. የተፈጥሮ ዑደት  2. በበሽታ የሚመጣ  3. የፈጣሪ እርግማን  4. አላዉቅም  5. ሌላ ካለ (ይገለፅ)---- |  |
| 202 | | የወር አበባ መንስኤ ምንድን ነዉ? | | 1. ሆርሞን  2. በኋጢያት የሚመጣ  3. የፈጣሪ እርግማን  4. በበሽታ የሚመጣ  5. አላዉቅም  6. ሌላ ካለ (ይገለፅ)---- |  |
| 203 | | \| የወር አበባ የሚመጣው ከየትኛው የሰውነት ክፍል ነው? \| \| --- \| | | 1. ከማኅፀን  2. ከረቤዛ  3. የሽንት ፊኛ  4. ሆድ  5. አላዉቅም  6. ሌላ ካለ (ይገለፅ)---፟ |  |
| 204 | | መደበኛ እና ጤነኛ የወር አበባ አንዴ ከመጣ ለምን ያክል ግዜ (ለስንት ግዜ) ይቆያል? | | 1. ከሁለት ቀናት ያነሰ  2. ከሁለት እስከ ሰባት ቀናት  3. ከሰባት ቀናት በላይ  4. አላውቅም |  |
| 205 | | አንድት ጤነኛ ልጃገረድ መደበኛ የወር አበባ በየ ስንት ቀኑ ታያለች? | | 1. ከ 20 ቀናት ያነሰ  2. ከ 20 እስክ 35 ቀናት  3. ከ35 ቀናት በላይ  4. አላውቅም |  |
| 206 | | \| የወር አበባ ኡደት እድሜ ልክ ይኖራል ብለሽ ታስቢያለሽ? \|  \| \| --- \| --- \| | | 1. አዎ  2. አይደለም |  |
| 207 | | \| የወር አበባ ንጹህ ያልሆነ እንደሆነ ታዉቂያለሽ? \| \| --- \| | | 1. አዎ  2. አይደለም |  |
| 208 | | የወር አበባ መጥፎ ጠረን እንዳለዉ ታዉቂያለሽ? | | 1. አዎ  2. አይደለም |  |
| 209 | | በወር አበባ ምክናየት ከትምህርት ቤት ቀርተሽ ታውቂያለሽ? | | 1 አወ  2 አላውቅም | ለ ጥያቄ ቁጥር 209 መልስዎ አላውቅም ከሆነ ወደ 211 ይሂዱ |
| 210 | | ለ ጥያቄ ቁጥር 209 መልስዎ አዎ ከሆነ,  በወር አበባ ወቅት ከትምህርት ቤት የምትቀሪበት ዋናው ምክናየትሽ ምንድን ነው (ከአንድ በላይ መልስ ይቻላል)? | | 1 ሽታው ስለ ሚያሳፍረኝ፣ ስለሚያናድደኝ እና ልብሴን ስለሚነካብኝ  2 ስለሚያመኝ  3 ለመጸዳጃ የሚሆን ዉሃ/ ምቹ ቦታ ስለለ  4 ለንጽህና የተጠቀምንበትን ቁስ መቀየሪያ ንጹህ ሽንት ቤት ስለለ  5 ትምህርት ቤት ውስጥ ንጽህና መጠበቂያ ልብስ ስለለ |  |
| 211 | | የመጀመሪያ የወር አበባ ከማየትሽ በፊት ስለ የወር አበባ ሰምተሽ ታውቂያለሽ? | | 1 አወ  2 አላውቅም | ለ ጥያቄ ቁጥር 211 መልስዎ አላውቅም ከሆነ ወደ 213 ይሂዱ |
| 212 | | ለ ጥያቄ ቁጥር 211 መልስዎ አዎ ከሆነ, የመረጃ ምንጭወት ምን ነበረ? | | 1. ከናቴ  2. ከመምህራን  3. ከጤና ባለሙያ  4. ከመገናኛ ብዙሃን (ሬድዮ/ ቴሌ ቪዥን)  5. ሌላ ካለ (ይገለፅ)---- |  |
| 213 | | ስለ የሰወች የመራቢያ የሰውነት ክፍል ኢንፌክሽን ወይም ስለ አባላዘር በሽታ ያውቃሉ? | | 1. አወ  2. አላውቅም |  |
| 214 | | ገበያ ላይ ያሉ የወር አበባ የንፅህና መጠበቂያዎችን ያውቃሉ? | | 1. አወ  2. አላውቅም |  |
| 215 | | ስለ ወር አበባ ጉዳይ ከወላጆችዎ ወይም ከጓደኛዎ ጋር በግለጽ ይወያያሉ? | | 1. አዎ  2. አንወያይም | ለ ጥያቄ ቁጥር 215 መልስዎ አንወያይም ከሆነ ወደ 217 ይሂዱ |
| 216 | | ለጥያቄ ቁጥር 215 መልስዎ "አዎ" ከሆነ በየትኞቹ ርእሶች / ጉዳዮች ላይ? | | 1.ስለ የወር አበባ የንጽህና አጠባበቅ.  2. ስለ የወር አበባ የንጽህና መጠበቂያ ዘዴዎች አጠቃቀም  3. ሁለቱም  4. ሌላ ካለ (ይገለፅ)---- |  |
| 217 | | ለጥያቄ ቁጥር 215 መልስዎ "አይደለም" ከሆነ, ለምን? | | 1. አሳፋሪ ስለሆነ  2. የተለመደ ስላልሆነ.  3. ምስጢራዊ ስለሆነ.  4.ሌላ ካለ (ይገለጽ)----- |  |
| **ክፍል 3 - በወር አበባ ጊዜ** **ንጽህና አጠባበቅ ትግበራ ጋር የተያያዙ ጥያቄዎች** | | | | | |
| 301 | | በወር አበባ ጊዜ የንጽህና መጠበቂያ ቁሳቁስ ይጠቀማሉ? | 1. አዎ  2. አልጠቀምም | | ለ ጥያቄ ቁጥር 301 መልስዎ አልጥቀምም ከሆነ ወደ 303 ይሂዱ |
| 302 | | ለ ጥያቄ ቁጥር 301 መልስዎ አዎ ከሆነ በወር አበባቸው ወቅት ምን አይነት የንጽህና መጠበቂያ ቁሳቁስ ይጠቀማሉ? | 1. ለአንድ ጊዜ ብቻ የሚያገለግሉ የወር አበባ መጠበቂያ(ሞዴስ).  2. ለአንድ ጊዜ ብቻ የሚያገለግል ቁርጥራጭ ልብስ  3.ለብዙ ጊዜ የሚያገለግሉ የወር አበባ መጠበቂያ (ሞዴስ).  4.የመጸዳጃ ወረቀት  5. የውስጥ ልብስ.  6. ሌላ ፡ ይገለጽ-------- | |  |
| 303 | ለአንድ ጊዜ ብቻ የሚያገለግሉ የወር አበባ መጠበቂያ(ሞዴስ) የማይጠቀሙበት ምክንያት ምንድን ነው? | | 1. ስለማላውቅ  2. መግዛት ስለማልችል  3. በቅርብ ስለማይገኝ  4. ስለማፍር  5.ሌላ ካለ ይገለጽ ---- | |  |
| 304 | በወር አበባ ጊዜ አባላዘርዎን (ብልትዎን) ይታጠባሉ? | | 1. አዎ  2. አልታጠብም | | ለ ጥያቄ ቁጥር 304 መልስዎ አልታጠብም ከሆነ ወደ 307 ይሂዱ |
| 305 | ለጥያቄ ቁጥር 304 መልስዎ አዎ ከሆነ ለማጽዳት ምን ይጠቁማል? | | 1. ውሃ ብቻ.  2. ሳሙና ውሃ.  3. ሌላ ፡ ይገለጽ -------- | |  |
| 306 | ለጥያቄ ቁጥር 304 መልስዎ አዎ ከሆነ በየቀኑ ለምንያክል ጊዜ ብልትዎን ይታጠባሉ? | | 1. አንድ ጊዜ  2. ሁለት ጊዜ  3. ሶስት ጊዜ  4. አራት ጊዜ እና ከዚያ በላይ | |  |
| 307 | በወር አበባ ጊዜ ገላውን ከሁልጊዜው በተለየ ይታጠባሉ? | | 1 አዎ  2.የለም | | ለ ጥያቄ ቁጥር 307 መልስዎ የለም ከሆነ ወደ 309 ይሂዱ |
| 308 | ለጥያቄ ቁጥር 307 መልስዎ አዎ ከሆነ በቀን ለስንት ጊዜ ይታጠቡ? | | 1. በቀን ሁለት ጊዜ እና በታች  2. በቀን ከሁለት ጊዜ በላይ | |  |
| 309 | የወር አበባ ንጽህና መተበቂያ ቁሳቁስዎን ይቀይራሉ? | | 1. አዎ  2. አልቀይርም | | ለ ጥያቄ ቁጥር 309 መልስዎ አልቀይርም ከሆነ ወደ 311 ይሂዱ |
| 310 | በየቀኑ የሚጠቀሙትን ቁሳቁስ ለምን ያህል ጊዜ ይቀይራሉ? | | 1. አንድ ጊዜ  2. ሁለት ጊዜ  3. ሦስት ጊዜ  4. ከሦስት ጊዜ በላይ | |  |
| 311 | የወር አበባ ንጽህና መጠበቂያዎን ከተጠቀሙ በኋላ የተጠቀሙትን ቁሳቁስ እንዴት ያስወግዳሉ? | | 1. ባገኘሁበት ቦታ  2. መፀዳጃ ቤት  3. የቆሻሻ ማጠራቀሚያ  4. ሌላ ይግለጹ--------- | |  |
| 312 | አዲሶቹን እና/ ወይም መልሰው የሚጠቀሙትን የንጽህና መጠበቂያ ቁሳቁስዎን የት ነው የሚያስቀምጡት? | | 1.መሳቢያ ውሰጥ  2. ቁም ሣጥን  3. መታጠቢያ ክፍል  4. ከዘወትር ልብሶች ጋር  5. አላስቀምጥም  6. ሌላ ይግለጹ---- --- | |  |
| 313 | መልሰው የሚጠቀሙበትን የንጽህና መጠበቂያ ቁሳቁስዎን ለማጽዳት ምን ይጠቀማሉ? | | 1. በሳሙና እና በውሃ  2. በውኃ ብቻ  3. ሌላ ይግለጹ---- --- | |  |
| 314 | መልሰው የሚጠቀሙባቸውን የንፅህና መጠበቂያዎች ካጠቡ በኋላ ለማድረቅ የት ያሰጣሉ? | | 1. ከቤት ውጭ ጥላ ውስጥ  2. ከቤት ውስጥ ጥላ ውስጠ  3. ቤት ውስጥ ፀሐይ ላይ  4. ውጭ ላይ በፀሐይ ብርሃን  5. ውጭ ላይ በሌሎች ልብሶች ተጋርዶ  6. ሌላ ስወር ቦታ  7. ሌላ ይግለጹ---- | |  |
| **ክፍል 4: ከትምህርት ቤት ቁሳቁሶች እና ከአካባቢው ጋር የተያያዙ ጥያቄዎች** | | | | | |
| 401 | ት/ቤት ውስጥ ስለወር አበባ ንጽህና አጠባበቅ ተምረው ያውቃሉ? | | 1. አወ  2. አልተማርኩም | |  |
| 402 | ትምህርት ቤቱ የውኃ አቅርቦት አለው? | | 1. አወ  2. የለውም | | ለጥያቄ ቁጥር 402 መልስዎ የለውም ከሆነ ወደ 404 ይሂዱ |
| 403 | ለጥያቄ ቁጥር 402 መልስዎ አዎ ከሆነ የውኃ አቅርቦት የሚያገኙት ከየት ነው | | 1= ከቧንቧ ወይም ጥልቅ ጉድጓድ በፓምፕ ወይም በደንብ የተቆፈረ ጉድጓድ  2=ከወንዝ ወይም ንጽህናውን ያልተጠበቀ ጉድጓድ | |  |
| 404 | ትምህርት ቤቱ የመጸዳጃ ቤት አለው? | | 1. አወ  2. የለውም | | ለጥያቄ ቁጥር 404 መልስዎ የለውም ከሆነ ወደ 408 ይሂዱ |
| 405 | ለጥያቄ ቁጥር 404 መልስዎ አዎ ከሆነ ምን አይነት የመፀዳጃ ቤት ነው ያለወ? | | 1= በውሃ ግፊት የሚሰራ የተሻሻለ መፀዳጃ ቤት  2= ክፍት ጉድጓድ ወይም ምንም (ሜዳ ላይ) | |  |
| 406 | ለጥያቄ ቁጥር 404 መልስዎ አዎ ከሆነ የሴቶች እና የወንዶች መፀዳጃ ቤቶች በተቃራኒ አቅጣጫዎች ናቸው? | | 1. አወ  2. አይደሉም | |  |
| 407 | ለጥያቄ ቁጥር 404 መልስዎ አዎ ከሆነ የሴት መጸዳጃ ቤት ክፍሎች ከውስጥ የሚቆለፉ ናቸው? | | 1.አወ  2. አይደሉም | |  |
| 408 | ትምህርት ቤት ውስጥ የወር አበባ ንፅህና መጠበቂያዎን የሚቀይሩበት ሚስጥራዊ ቦታ አለ? | | 1. አወ  2. የለም | |  |
| 409 | ትምህርት ቤት ውስጥ የሴቶች የገላ መታጠቢያ የተለየ ነው? | | 1.አወ  2. አይደለም | |  |
