# Supplementary material for: Menstrual hygiene management knowledge, practice and associated factors Among School Girls, Northeast Ethiopia
Source: PLoS One. 2022 Jul 19;17(7):e0271275. doi: 10.1371/journal.pone.0271275 (PMC9295965; doi:10.1371/journal.pone.0271275)
Supplement: S2 Appendix — A survey on Menstrual Hygiene management knowledge, practice and associated factors Among School Girls, Northeast Ethiopia. (DOCX) [file pone.0271275.s002.docx]

Annexe IV English Version Questionnaire

Addis Ababa University; college of health science; school of Nursing and Midwifery Department of Midwifery; Questionnaires for assessment of the knowledge, practice, and associated factors with menstrual hygiene management among Mekdela secondary school girls.

| **Part 1: -Socio-demographic, Economic and Family related questions** | | | | | |
| --- | --- | --- | --- | --- | --- |
| S. No | **Questions** | | Possible answer | | Code |
| 101 | How old are you now? | | I am ---------years old | |  |
| 102 | Your grade level | | 1. =9^th^ 2. =10th  3. =11^th^ 4. =12^th^ | |  |
| 103 | How old were you at your menarche? | | I was --------years old | |  |
| 104 | Your residence | | 1. Urban  2. Rural | |  |
| 105 | Your religion | | 1. Orthodox  2. Muslim  3. Protestant  4. Catholic  5. Others (specify) ----- | |  |
| 106 | Your marital status | | 1. Single  2. Divorced  3. Widowed  4. Married  5. Not applicable | |  |
| 107 | With whom do you live? | | 1. Both parents  2. Mother only  3. Relatives  4. Father Only  5. Others (specify) ---- | |  |
| 108 | What is your mother’s educational status? | | 1. No formal education  2. Primary  3. Secondary  4. College and above | |  |
| 109 | What is your father’s educational status? | | 1. No formal education  2. Primary  3. Secondary  4. College and above | |  |
| 110 | What is your mother’s occupational status? | | 1. Housewife  2. Student  3. Merchant  4. Private organization employee  5. Governmental employee  6. Daily laborer  7. Others (specify) ----- | |  |
| 111 | What is the father’s occupational status? | | 1. Farmer  2. Merchant  3. Governmental employee  4. private organization employee  5. Daily laborer  6. Driver  7. Others (specify) ----- | |  |
| 112 | Do your parents provide permanent pocket money regularly? | | 1. Yes  2. No | | If your answer is No go to Q- 114 |
| 113 | If your answer to question No 112 is “Yes”, what is your monthly income? | | 1. </= 200  2. (200-400)  3. >/= 400 | |  |
| 114 | Do your parents have a private shower? | | 1=yes  0 =No | |  |
| **Part 2: -Menstrual hygiene management knowledge related questions** | | | | | |
| 201 | What is menstruation? | 1. Is a physiological process  2. Is a pathological process  3. I curse from god  4. I don’t know  5. Other (specify) | |  | |
| 202 | What is the cause of menstruation? | 1. Hormone.  2. Is caused by sin.  3. It is a curse of God.  4. Is caused by a disease.  5. I don’t know.  6. Other (specify) | |  | |
| 203 | From which organ does the menstrual blood come? | 1. Uterus  2. Vagina  3. Urinary bladder  4. Abdomen  5. I don’t know  6. Other (specify) | |  | |
| 204 | How long is the normal menstrual bleeding duration? | 1. <2 Days.  2. 2-7 Days  3. >7 Days  4. Don’t know | |  | |
| 205 | What is the normal duration of the menstrual cycle? | 1. <20 Days  2. 20-35 Days  3. >35 Days  4. Don’t know | |  | |
| 206 | Do you Know that Menstruation is a lifelong process? | 1. Yes  2. No | |  | |
| 207 | Do you know that menstrual blood is unhygienic? | 1. Yes  2. No | |  | |
| 208 | Do you know that it is foul-smelling during menstruation? | 1. Yes  2. No | |  | |
| 209 | Ever missed school because of menstruation? | 1. Yes  2. No | | If your answer is No go to Q- 211 | |
| 210 | If your answer is ‘Yes’ for question No 209 main reasons for missing school (Multiple answers) | 1. Afraid of odor/being teased/ staining clothes  2. Pain  3. Lack of water/place a convenient place to wash  4. Unclean/no latrine to change pad  5. No access to pads or cloths in school | |  | |
| 211 | Have you heard about menstruation before menarche? | 1.Yes  2. No | | If your answer is No go to Q- 213 | |
| 212 | If your answer is ‘Yes’ for question No 211, what is the source of information? | 1. Mother.  2. Teacher.  3. Health personnel.  4. Mass media (radio/ TV).  5. others; specify----- | |  | |
| 213 | Do you know about RTIs/ STIs? | 1. Yes  2. No | |  | |
| 214 | Do you know sanitary pads in the market? | 1. Yes  2. No | |  | |
| 215 | Do you freely discuss menstruation issues with your parents, friends? | 1. Yes  2. No | | If your answer is No go to Q- 217 | |
| 216 | If your answer to question No 215 is “Yes”, in what topics/issues why? | 1. About menstrual hygiene management.  2. About methods on how to use sanitary pads.  3. both  4. other, specify------ | |  | |
| 217 | If your answer to question No 115 is “No”, why? | 1.Because of shamefulness  2. Not habitual.  3. Privacy.  4.other specify-------- | |  | |
| **Part 3: -Menstrual hygiene practice related questions** | | | | | |
| 301 | Do you use sanitary material(s) during menstruation? | | 1. Yes  2. No | |  |
| 302 | If your answer is Yes for Q no 301,  what sanitary material do you use during menstruation? | | 1. Disposable sanitary pads.  2. Disposable piece of rags.  3. Reusable sanitary pads  4. Paper/toilet paper.  5. Underwear.  6. Others; specify----- | |  |
| 303 | What is your reason for not using a disposable sanitary pad? | | 1. Lack of knowledge  2. High cost  3. Unavailability  4. Shyness  5. Others; specify----- | |  |
| 304 | Do you wash your genitalia during menstruation? | | 1 Yes  2 No | | If your answer is No go to Q- 307 |
| 305 | If your answer for question no 304 is yes what medium do you use for your genital cleaning purpose? | | 1. Only Water.  2. Soap and water.  3. Others; specify-------- | |  |
| 306 | If your answer to question no 304 is yes how often do you wash your genitalia per day? | | 1. Once  2. Twice  3. Thrice  4. >=Four times. | |  |
| 307 | Do you take bath during menstruation (exceptional from the usual)? | | 1. Yes  2. No | | If your answer is No go to Q- 309 |
| 308 | If your answer to question No 307 is yes how often do you take bath during menstruation per day? | | 1. <= Two times in a day.  2. > Two times in a day | |  |
| 309 | Do you change your sanitary material(s) during menstruation? | | 1. Yes  2. No | | If your answer is No go to Q- 311 |
| 310 | How often do you change absorbent material per day? | | 1. Once  2. Twice  3. Three times  4. More than three times | |  |
| 311 | How do you dispose of menstrual materials after use? | | 1. Open field  2. Latrine  3. Put in the bin  4. Others; specify ------- | |  |
| 312 | Where do you store your new and/or reusable absorbent(s)? | | 1. Drawers  2. Dress cabinet  3. Bathrooms  4. Store with routine cloth  5. Don’t store  6. Others; specify-- | |  |
| 313 | What materials do you use for Washing of the reusable cloth? | | 1. With soap and water  2. With water, only  3. Others (specify) ---------- | |  |
| 314 | Where do you put/ keep your reusable sanitary pads after washing for drying? | | 1. In the shade outside  2. In the shade inside  3. In the sunlight inside  4. In the sunlight outside  5.Hidden under other clothes  6. Hidden elsewhere  7 Other; specify------------ | |  |
| **Part 4; School facility or Environmental related Questions** | | | | | |
| 401 | Have you ever learned about menstrual hygiene management in the school? | | 1. Yes  2. No | |  |
| 402 | Does the school have a water source? | | 1. Yes  2. No | | If your answer is No go to Q- 404 |
| 403 | If your answer is ‘Yes’ for question No “402” what is the source of water? | | 1= piped into a dwelling or  borehole with a pump or  protected dug well  2= pond or unprotected well | |  |
| 404 | Does the school have a toilet facility? | | 1. Yes  2. No | | If your answer is No go to Q- 406 |
| 405 | If your answer is ‘Yes’ for question No “404’’ what type of toilet facility is there? | | 1= flush or ventilated  improved latrine  2= open pit or none  (bush field) | |  |
| 406 | Does the school have females and males’ toilets in opposite directions? | | 1. Yes  2. No | |  |
| 407 | Does the school have females’ toilets kept locked inside? | | 1. Yes  2. No | |  |
| 408 | Does the school have private space to manage period at school? | | 1. Yes  2. No | |  |
| 409 | Does the school have a separate bathroom for girls? | | 1. Yes  2. No | |  |

Thank you very much for your patience

Data collector’s name--------------signature-------------- Date -------------
